# An Actinic Keratosis Auxiliary Diagnosis Method Based on an Enhanced MobileNet Model

**DOI:** 10.3390/bioengineering10060732

**Published:** 2023-06-19

**Authors:** Shiyang Li, Chengquan Li, Qicai Liu, Yilin Pei, Liyang Wang, Zhu Shen

**Affiliations:** 1 School of Information Science and Engineering, Yunnan University, Kunming 650091, China; lishiyang_gflv@stu.ynu.edu.cn; 2 School of Clinical Medicine, Tsinghua University, Beijing 100084, China; licq19@mails.tsinghua.edu.cn (C.L.); pyla02588@btch.edu.cn (Y.P.); 3 Center for Reproductive Medicine, The First Affiliated Hospital of Fujian Medical University, Fuzhou 350004, China; liuqc22@mails.tsinghua.edu.cn; 4 Vanke School of Public Health, Tsinghua University, Beijing 100084, China; 5 Department of Dermatology, Guangdong Provincial People’s Hospital (Guangdong Academy of Medical Sciences), Southern Medical University, Guangzhou 510080, China

**Keywords:** actinic keratosis, enhanced MobileNet, auxiliary diagnosis, model comparison

## Abstract

Actinic keratosis (AK) is a common precancerous skin lesion with significant harm, and it is often confused with non-actinic keratoses (NAK). At present, the diagnosis of AK mainly depends on clinical experience and histopathology. Due to the high difficulty of diagnosis and easy confusion with other diseases, this article aims to develop a convolutional neural network that can efficiently, accurately, and automatically diagnose AK. This article improves the MobileNet model and uses the AK and NAK images in the HAM10000 dataset for training and testing after data preprocessing, and we performed external independent testing using a separate dataset to validate our preprocessing approach and to demonstrate the performance and generalization capability of our model. It further compares common deep learning models in the field of skin diseases (including the original MobileNet, ResNet, GoogleNet, EfficientNet, and Xception). The results show that the improved MobileNet has achieved 0.9265 in accuracy and 0.97 in Area Under the ROC Curve (AUC), which is the best among the comparison models. At the same time, it has the shortest training time, and the total time of five-fold cross-validation on local devices only takes 821.7 s. Local experiments show that the method proposed in this article has high accuracy and stability in diagnosing AK. Our method will help doctors diagnose AK more efficiently and accurately, allowing patients to receive timely diagnosis and treatment.

## 1. Introduction

Actinic keratosis (AK) is a skin lesion related to ultraviolet (UV) radiation, mainly affecting skin areas exposed to sunlight for a long time, such as the face, scalp, neck, forearms, and backs of the hands. The diseased part of the skin shows keratosis proliferation, presenting symptoms such as rough skin, pigmentation, and scales. AK mainly occurs in the middle-aged and elderly population, especially Caucasians, and its incidence is related to multiple factors such as the dose of ultraviolet radiation, genetic factors, and immune status [1,2]. It is one of the most common precancerous skin lesions, presenting as reddish-brown or yellow spots or small patches with clear boundaries, and about 10% of patients may develop into squamous cell carcinoma (SCC). Therefore, early diagnosis and timely treatment of AK are of great significance [3,4,5]. The current treatment methods for AK include cryotherapy, drug therapy, photodynamic therapy, laser therapy, etc. Early detection and treatment of AK can effectively reduce the risk of cancer in patients and improve their quality of life [6].

Currently, the diagnosis of AK is difficult and can easily be confused with other non-actinic keratoses (NAK) such as seborrheic keratosis and geriatric skin keratosis [7,8,9,10]. Traditional diagnostic methods mainly rely on doctors’ naked eye observation, clinical experience, and pathological examination. These methods are costly and may be traumatic, and the diagnosis results may be influenced by subjective factors. Therefore, developing an efficient, automatic, and accurate diagnosis method for AK is of great clinical significance.

Machine learning technology provides a new way for the auxiliary diagnosis of skin diseases. With the development of computer vision, the application of image recognition based on deep learning in medical diagnosis is increasing, involving fields such as cancer recognition, tissue cell classification, and tumor image segmentation. Kawahara et al. [11] used convolutional neural networks (CNNs) to classify skin pathology images, obtaining good results. Kaur et al. [12] proved that the classification accuracy of the hybrid deep learning method in six kinds of skin images is better than that of traditional CNN models. Ferguson et al. [13] proposed a CNN network structure based on mask regions with high recognition accuracy. Harangi et al. [14] used the integrated output of the softmax layer of four different neural architectures for intelligent diagnosis of melanoma. In general, the mainstream method of using machine learning to recognize skin diseases is deep CNN. These networks are characterized by long training times and high computational requirements; thus, they have significant limitations in actual clinical applications.

However, there is very little research specifically targeting keratosis. In fact, the main research currently is the shallow neural network method AK-DL proposed by Wang L et al. [15] in 2020. This is a shallow neural network model that has superior performance in diagnosing keratosis compared with deep neural networks. However, despite the excellent performance of the model in various indicators, its accuracy needs to be improved. Therefore, this study aims to develop a high-accuracy, efficient, and low-device-requirement AK image recognition method based on shallow CNN to improve clinical diagnostic efficiency and facilitate self-monitoring and prevention of skin cancer.

**Figure 1:**
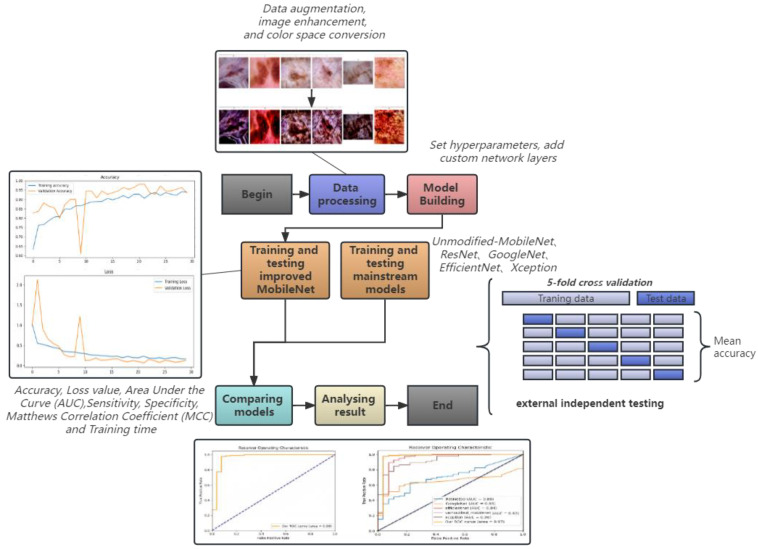
The workflow of the improved MobileNet model.

In this study, we propose an improved MobileNet for the auxiliary diagnosis of AK. Based on the traditional MobileNet, we introduce a custom preprocessing function, apply Lab Space and Contrast Limited Adaptive Histogram equalization to the input image, modify the network architecture of MobileNet, add custom network layers, and use five-fold cross-validation to improve the stability and generalization ability of the model. An essential aspect of our study was the implementation of an external validation strategy, using a separate dataset from The International Skin Imaging Collaboration (ISIC). This validation served a dual purpose; to verify the broad applicability of our preprocessing method and to assess the generalization capability of our model on previously unseen data. This rigorous validation strategy further solidifies the credibility of our proposed model, demonstrating its robustness and adaptability to different data contexts. To highlight the superiority of our model, we compared it with the base model MobileNet and four other mainstream models (ResNet, GoogleNet, EfficientNet, and Xception). The test results show that our model is highly accurate and fast and is expected to be applied to clinics in the future by embedding our model into portable AK devices. The workflow of this study is shown in Figure 1.

Overall, the highlights of this paper are as follows:

1. We propose and implement an improved MobileNet model. In response to the characteristics of actinic keratosis, we optimized the network structure of the model to better capture subtle differences in skin images. At the same time, we introduced appropriate activation and loss functions to further enhance the performance of the model.
2. Although there have been some studies exploring deep learning methods for the diagnosis of skin diseases, there is little research specifically targeting the identification of actinic keratosis. Against this background, this study helps to fill the gap in the re-search of actinic keratosis identification, thus improving the effects of recognizing actinic keratosis.
3. In the data preprocessing stage, we used the LAB color space to replace the traditional RGB color space and used other image processing techniques, such as image enhancement and segmentation, to further improve the performance of the model.
4. We made use of external validation to ascertain the broad adaptability of our data preprocessing method and the generalization ability of our model. By testing our model with a distinct dataset, we demonstrated its capability to perform excellently in the face of unseen data, thus showing the robustness of our model in various data scenarios. This aligns with the real-world clinical setting, where diverse and varied data inputs are common. This validation step underscores the potential of our model for practical deployment and its suitability for integration into future clinical workflows.

## 2. Materials and Methods

### 2.1. Data Sources

The dataset used in this study comes from the Harvard University Dataverse platform, with the download link: https://www.kaggle.com/datasets/kmader/skin-cancer-mnist-ham10000, accessed on 21 September 2018. The dataset, named HAM10000, was jointly collected and collated by the Department of Dermatology of the University of Vienna, Austria, and the Cliff Rosendahl Skin Cancer Practice in Queensland, Australia, covering skin disease images over 20 years. HAM10000 contains 100,015 images of seven common skin diseases, all strictly confirmed by dermatology experts [16]. In this study, we selected a part of the data for analysis, including 327 skin images diagnosed as keratosis (AK) and 1099 skin images diagnosed as none-actinic keratoses (NAK). At the same time, based on the dataset, we collected relevant clinical information and data of the patients, such as gender, location of onset, and age group of onsets.

To validate the generalizability and novelty of our method, we employed an external dataset released by ISIC in 2019 (https://challenge.isic-archive.com/data/#2019, accessed on 16 August 2019) as an external validation set. The dataset comprises 1011 AK images, of which only 867 were available for use in training data. The remaining images were unavailable as they were being utilized in live challenges. Similarly, only 2624 out of the entire Benign Keratosis-like Lesion (BKL) image collection were available due to the same circumstances. After removing images that coincided with those in the original HAM10000 dataset, we used the remaining 540 AK images and 1525 BKL images for external independent testing. Our use of this external dataset not only strengthens our model’s robustness but also enhances its credibility by ensuring that our model is evaluated against unseen, non-overlapping data, thereby effectively demonstrating its real-world applicability.

To our knowledge, there is a considerable gap in research that only analyzes keratosis images from the ISIC dataset. Currently, the main research is the binary auxiliary diagnostic method for actinic keratosis proposed by Wang L et al. in 2020. Therefore, our research is very necessary.

### 2.2. Data Preprocessing

In this research, our initial step involved preprocessing the images to optimize model performance. The process encompassed transformations such as color space conversion, histogram equalization, and data augmentation. To begin with, we transitioned images from the RGB color space to the Lab color space, a change intended to extend the color gamut. Lab space is characterized by a wider range compared with RGB and CMYK color modes, which aids in rectifying the uneven distribution prevalent in these modes [17]. Additionally, lab space provides the distinct advantage of separating brightness from color, which simplifies the adjustment process and facilitates easier handling by the computer [18]. Subsequent to this color conversion, we employed the Contrast Limited Adaptive Histogram Equalization (CLAHE) method to the brightness channel. CLAHE is designed to enhance the local contrast of the image by constraining the amplification of contrast, thereby improving the image quality. This method is particularly effective in highlighting textured areas of keratosis, providing the model with a more discernible identification of skin cancer image features [19,20].

Acknowledging the existing imbalance in our dataset, we implemented data augmentation to bolster the model’s robustness and foster its generalization capability, effectively reducing the risk of overfitting [21]. Utilizing the ImageDataGenerator class, we performed operations including rotation, translation, scaling, horizontal flipping, and vertical flipping on the images [22]. These manipulations enabled us to generate novel image variants from the existing data, thereby supplementing our training dataset. Furthermore, each parameter involved in the data augmentation process can be adjusted as needed, paving the way for optimal model performance. In summary, our preprocessing method provides a robust foundation for enhancing the model’s ability to accurately classify skin cancer images.

We were conscious of potential issues related to the application of our preprocessing steps on specific data, which may not operate correctly when extended to other datasets. To address this, we sought to verify the efficacy and compatibility of our preprocessing methodology with external data.

### 2.3. Model Architecture

#### 2.3.1. Our Model—Enhanced MobileNet

In this study, we adopted MobileNet as the base model and made improvements on it. MobileNet is an efficient deep neural network designed for mobile devices and embedded visual applications, and its core is based on depthwise separable convolution, including 28 convolution layers. Compared with traditional convolution layers, depthwise separable convolution significantly reduces computational complexity and the number of parameters, thus achieving a more compact model architecture and shorter training time, making it very suitable for deployment on resource-limited devices [23]. However, due to the limitations of MobileNet, such as relatively low accuracy and limited robustness to changes in input data [24], it is urgent to develop an improved method to overcome these limitations while maintaining efficiency.

**Figure 2:**
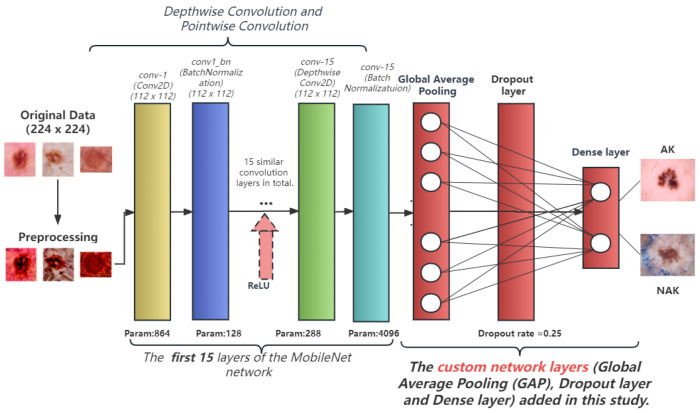
The network architecture of the improved MobileNet model.

To enhance the performance of MobileNet, we made a series of modifications to its original architecture. First, we introduced custom data preprocessing, applying Lab Space and Contrast Limited Adaptive Histogram Equalization (CLAHE) to the input images to enhance image contrast and detail performance, thereby helping the model learn and recognize features more easily. Our model accepts input images of size (224, 224, 3). We adapted the original 28-layer MobileNet architecture to a more compact and efficient 15-layer structure, where each layer employs depthwise separable convolution followed by Batch Normalization and a ReLU activation function. This modification substantially reduces the complexity of the model, greatly improves the computation speed, and mitigates the risk of overfitting. Traditional CNN models have certain deficiencies in generalization ability, leading to overfitting in the case of insufficient data. Considering the relatively small number of keratosis images, we introduced a dropout layer to prevent overfitting. During training, dropout can discard some neurons in the hidden layer with a certain probability, causing changes in the network structure. The entire process of dropout is equivalent to averaging multiple different neural networks. To some extent, it can also reduce the complex mutual adaptation between neurons, thus achieving a regularization effect. After several tests, we found that dropout set to 0.25 performed best. The final output layer is a fully connected layer with 2 output units and a softmax activation function, corresponding to the two categories of our problem: “AK” and “NAK”. This is used to highly purify the features extracted by the convolution layer, thereby mapping the distributed features to the sample space. Our model architecture is shown in Figure 2.

To improve the stability and generalization ability of the model, we also used 5-fold cross-validation. During training, we used the Adam optimizer with a learning rate of 0.01 and a categorical cross-entropy loss function. At the same time, we monitored some key performance indicators, including classification accuracy, precision, recall, and the Area Under the ROC Curve (AUC). During training, we adjusted the learning rate according to the accuracy on the validation set through defining a callback mechanism. Whenever the learning rate reached a stable level, we reduced it by half. In addition, we also used the ModelCheckpoint callback to save the model weights corresponding to the highest validation accuracy. After debugging, this model used the softmax function as the activation function. It can convert the output into a probability distribution, and each node in the output layer corresponds to the probability of a category. At the same time, compared with the sigmoid and ReLU functions, the softmax function has better gradient stability during backpropagation, thus reducing the problem of gradient vanishing or explosion. Moreover, although the softmax function involves exponential operations, the range of values involved in the calculation is relatively small, so the computational cost is relatively low.

#### 2.3.2. Comparative Models

In this paper, the improved model is compared with the base model MobileNet and four mainstream models (CPU: Intel Xeon E5-2680v3, USA, Motherboard: ASUS X99 server motherboard, China).

To address issues such as gradient vanishing or gradient explosion brought by the continuous deepening of the network, He and others [25] from Microsoft Research Asia proposed the Residual Network (ResNet) in 2015.

The core idea is to pass features skip-level to retain all original information, thereby reducing a large number of network parameters and improving network efficiency and performance [26]. In this study, we used the pre-trained ResNet50 model, removed the top fully connected layer, set the size of the input image to 224 × 224, and then added a global average pooling layer, a dropout layer, and a fully connected layer for classification at the output end of the ResNet50 model. The number of parameters is about 23.5 million. The GoogleNet model has a modular unit, Inception, which can increase the depth and width of the network without increasing computational complexity [27]. 

In this study, we used the pre-trained InceptionV3 network and made similar modifications to the top layer. GoogleNet has fewer parameters, mainly because it uses a large number of 1 × 1 convolution kernels in its design, reducing the number of parameters.

To highlight the superiority of this improved model, we also included the traditional unimproved MobileNet in the comparative model. 

MobileNet is an efficient deep neural network designed for mobile devices and embedded vision applications. Its core is depthwise separable convolution, including 28 convolutional layers. Compared with traditional convolutional layers, depthwise separable convolution significantly reduces computational complexity and the number of parameters, resulting in a more compact model architecture and shorter training time, making it very suitable for deployment on resource-limited devices. EfficientNet is a model developed by Google that can automatically adjust the network structure. 

By adjusting the depth, width, and resolution of the network, it can effectively reduce the complexity and computational load of the model while maintaining accuracy [28]. In this study, we used the pre-trained EfficientNet model and made similar modifications to the top. The Xception model is an improved model based on the Inception architecture. 

Its core idea is to separate the spatial convolution and cross-channel convolution in the convolutional layer. This separation can improve the performance and efficiency of the model while reducing the number of parameters [29].

### 2.4. Experiment

In this study, we meticulously crafted an internal and external validation strategy to robustly assess the performance of our model. For internal validation, we utilized a dataset consisting of 327 actinic keratosis (AK) images and 1099 BKL images. We adopted a five-fold cross-validation method for this process. In five-fold cross-validation, the original sample is randomly partitioned into five equal-sized subsamples. Of the five subsamples, a single subsample is retained as the validation data for testing the model, and the remaining four subsamples are used as training data. The cross-validation process is then repeated five times, with each of the five subsamples used exactly once as the validation data. The advantage of this method over repeated random sub-sampling is that all observations are used for both training and validation, and each observation is used for validation exactly once. This process is beneficial in mitigating overfitting, offering a more generalized model performance. For external validation, we used a separate dataset from ISIC. This dataset consisted of 540 AK images and 1525 BKL images (removed images that coincided with those in the original HAM10000 dataset). This was carried out to ensure our model’s effectiveness and adaptability on an unseen, independent dataset. External validation is crucial as it verifies the model’s applicability to real-world, previously unseen data. Moreover, data augmentation techniques such as random rotation, translation, scaling, and flipping were utilized to prevent overfitting. We used tensorflow’s ImageDataGenerator for data augmentation to obtain a more accurate model performance evaluation. 

During the modeling phase, we took MobileNet as the base model and made improvements. MobileNet is an effective lightweight deep convolutional neural network that primarily uses depthwise separable convolution to minimize computational costs, allowing the model to achieve high accuracy at a lower computational cost. Improvements include adding a global average pooling layer, a dropout layer, and a fully connected layer, using a softmax activation function, and conducting experimental tests to retain only the first 15 layers of the MobileNet model, tailoring it to best suit our task. During the training phase, we used the Adam optimizer and the cross-entropy loss function, set the initial learning rate to 0.01, and used the learning rate callback function ReduceLROnPlateau to dynamically adjust the learning rate. In selecting the optimal learning rate for the Adam optimizer during our model’s training phase, we deviated from conventional recommendations and opted for an initial learning rate of 0.01 rather than the typically suggested 0.001. This decision was not made lightly but was a consequence of numerous preliminary experiments and gradient tests that demonstrated the superiority of a 0.01 learning rate in our specific experimental context and with our particular dataset. A 0.01 learning rate facilitated a more efficient learning process, promoting faster convergence while maintaining stability. We fully appreciate the profound influence of learning rate selection on the accuracy of neural network classifiers, and as such, we conducted extensive experiments during the model development process to establish the ideal learning rate. In our trials, a learning rate of 0.01 enabled rapid initial descent in our model’s training phase, while also allowing for a judicious deceleration of learning speed as the solution neared optimality, thereby mitigating the risks of gradient vanishing and overfitting. When the model’s validation accuracy did not improve for 2 consecutive epochs, the learning rate was halved until it was reduced to 0.00001. This allowed the model to converge quickly in the initial stage of training and optimize the model parameters more meticulously in the later stage of training.

### 2.5. Experiment Evaluation

This paper employs a five-fold cross-validation approach to assess the model’s performance. In this approach, the dataset is partitioned into five equal parts. The model is trained on four parts (or ‘folds’), and the remaining part is used to validate the model. This process is repeated five times, with each of the five parts used exactly once for validation. The results of the five evaluations are then averaged to produce a single estimation. The results are also expressed as the mean value plus or minus the 95% confidence interval, providing an interval estimate of the model’s performance. This measure of uncertainty is significant because it conveys the statistical accuracy of the estimate.

The performance metrics employed in this paper include accuracy, loss value, AUC, sensitivity, specificity, and Matthews Correlation Coefficient (MCC). Each of these metrics measures different aspects of the model’s performance: Accuracy is the proportion of true results (both true positives and true negatives) in the total number of cases examined. It measures the overall correctness of the model. Loss Value is a measure of how well the model’s predictions match the actual values. Lower loss values indicate better performance. The AUC of the Receiver Operating Characteristic (ROC) curve is a comprehensive metric for evaluating binary classification models. An AUC of 1 represents a perfect model, while an AUC of 0.5 represents a model that is no better than random guessing. Sensitivity (also known as recall or true positive rate) measures the proportion of actual positive cases that are correctly identified by the model. Specificity (also known as true negative rate) measures the proportion of actual negative cases that are correctly identified by the model. MCC is a measure of the quality of binary classifications. It takes into account true and false positives and negatives and is generally regarded as a balanced measure which can be used even if the classes are of very different sizes. The MCC is in essence a correlation coefficient between the observed and predicted binary classifications; it returns a value between −1 and +1. A coefficient of +1 represents a perfect prediction, 0 represents no better than random prediction, and −1 indicates total disagreement between prediction and observation. These metrics together provide a holistic view of the model’s performance, each adding a unique perspective on the strengths and potential weaknesses of the model. By considering all these metrics, we can make a comprehensive and robust assessment of the model’s performance. The following is the calculation formula of each performance index.

(1) $$Acc=\frac{TN+TP}{TN+TP+FN+FP}$$

(2) $$Loss=-(y\times \log p+\left(1-y\right)\log\left(1-p\right)$$

(3) $$Sens=\frac{TP}{TP+FN}$$

(4) $$Spec=\frac{TN}{TN+FP}$$

(5) $$MCC=\frac{TP\times TN-FP\times FN}{\sqrt{\left(TP+FP\right)\left(TP+FN\right)\left(TN+FP\right)\left(TN+FN\right)}}$$

## 3. Results

### 3.1. Dataset Visualization and Its Preprocessing

**Figure 3:**
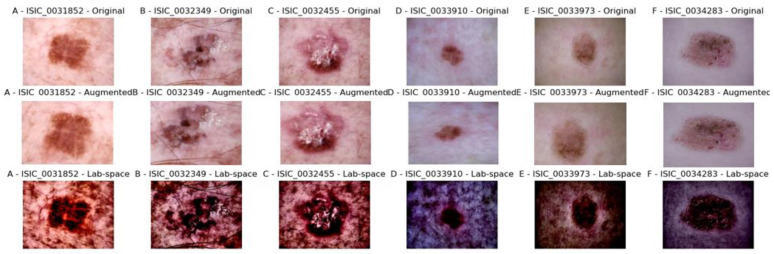
Comparisons of skin images before and after preprocessing. (**Top row**: original images of AK (**A**–**C**) and NAK (**D**–**F**). **Middle row**: augmented AK (**A**–**C**) and NAK (**D**–**F**) images. **Bottom row**: AK (**A**–**C**) and NAK (**D**–**F**) images post-spatial transformation. Each image is marked with its original number.).

In this study, we first preprocessed the images. Figure 3 is the visualization of the images after preprocessing. In the first row, A–C are the original images of AK, and D–F are the original images of NAK. In the second row, A–C are the AK images after data augmentation, and D–F are the NAK images after data augmentation. In the third row, A–C are the AK images after image spatial transformation, and D–F are the NAK images after image spatial transformation. Each image indicates its original number. The above processing not only improves the generalization ability of the model, mitigates overfitting, but also separates brightness from color, making it simple and easy for computer processing.

**Figure 4:**
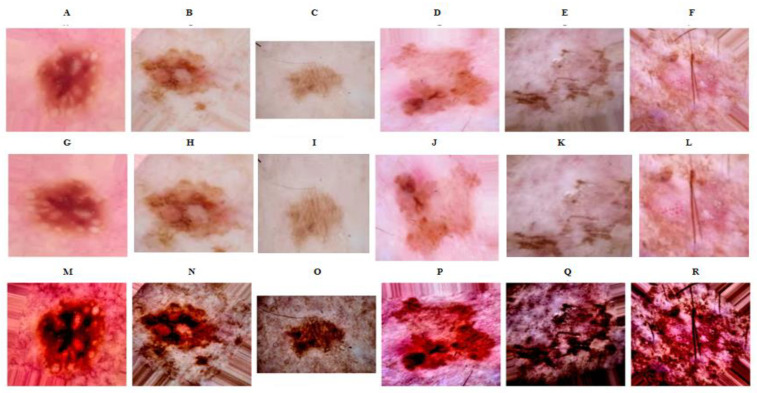
Comparisons of skin images before and after preprocessing on an external validation set. (**Top row**: original images of AK (**A**–**C**) and NAK (**D**–**F**). **Middle row**: augmented AK (**G**–**I**) and NAK (**J**–**L**) images. **Bottom row**: AK (**M**–**O**) and NAK (**P**–**R**) images after spatial transformation.).

Upon applying our preprocessing methods to the external dataset, we found that our methods could be effectively implemented. The images preprocessed from the ISIC 2019 dataset exhibited enhanced contrast, with features of skin lesions being noticeably highlighted, and thus easier for the model to identify. This validation with the ISIC dataset not only corroborates the effectiveness of our preprocessing methodology, but also underscores its adaptability to other datasets, hence enhancing its overall applicability in skin cancer detection tasks. Figure 4 depicts the enhancement in the image quality post our preprocessing steps.

### 3.2. Model Result

In this study, we proposed a deep learning model based on MobileNet for the identification of two different types of skin cancer: AK and NAK. The model demonstrated excellent accuracy and stability in a five-fold cross-validation, specifically an accuracy of 0.9265 ± 0.0336, loss value of 0.1732 ± 0.0041, and an AUC value of 10.97 ± 0.02, demonstrating stable performance across different thresholds. Notably, the model’s sensitivity and specificity were also quite high, reaching 0.9205 and 0.9242, respectively, indicating a strong ability to correctly identify both true positives and true negatives. Moreover, the model’s MCC value was 0.8840, a comprehensive metric considering true positives, true negatives, false positives, and false negatives, indicating superior performance in binary classification tasks.

**Figure 5:**
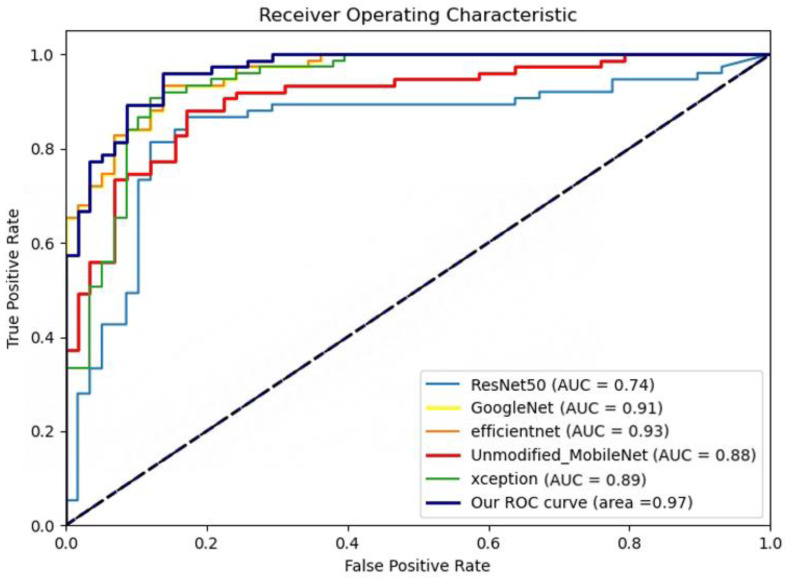
ROC curves of improved MobileNet and mainstream machine learning algorithms.

To compare the performance of our model with other mainstream models, we compared it with ResNet, GoogleNet, EfficientNet, Unmodified MobileNet, and XceptionNet. Our Modified MobileNet exhibited clear superiority, with performance indicators such as sensitivity, specificity, and MCC all surpassing those of the comparative models (Table 1), further validating its superiority in the task of skin cancer recognition. For instance, our model achieved an AUC value of 0.97, significantly higher than other models, demonstrating superior classification performance at different thresholds (the corresponding ROC curves are shown in Figure 5). Furthermore, our model showed a significant advantage in training time, totaling only 821.74 s, far less than the other models. For example, the training time of the slowest model, ResNet reached 3757.08 s, and the fastest comparative model, Unmodified MobileNet, required 1140.91 s. This implies that our model can achieve higher performance in a shorter time, making it more suitable for deployment in clinical applications, assisting physicians in faster and more accurate diagnosis of skin cancer, thereby improving patient treatment outcomes.

**Figure 6:**
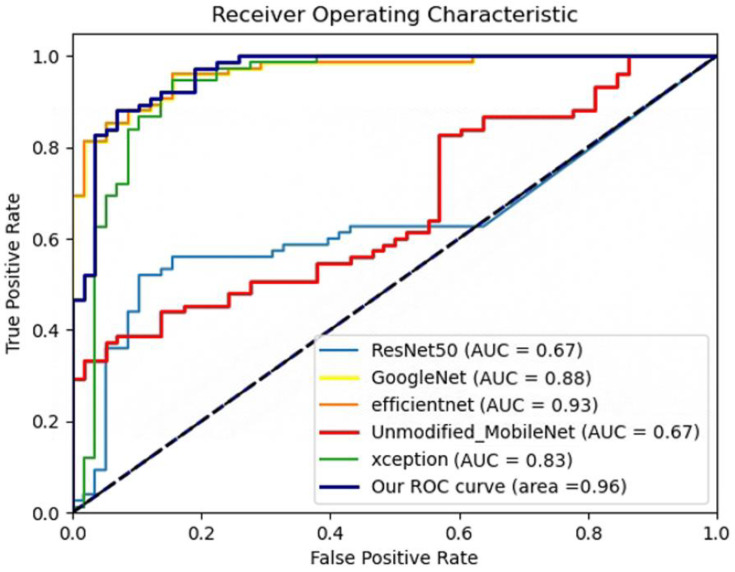
ROC curves of improved MobileNet and mainstream machine learning algorithms on external dataset on the external validation set.

Building upon our successful results, we also aimed to evaluate the performance of our model in an external independent testing environment, an integral aspect of validating its practical applicability and its capability to generalize. In this regard, we utilized an external dataset for this independent validation. The results of the external independent test reaffirmed the superior performance of our Modified MobileNet model. With an accuracy of 0.9197, sensitivity of 0.9088, specificity of 0.9246, and a MCC of 0.8575, our model convincingly outperformed other mainstream models such as Unmodified MobileNet, ResNet, GoogleNet, EfficientNet, and Xception, as can be observed in Table 2. Additionally, the corresponding ROC curves are shown in Figure 6.

The results of this independent testing clearly demonstrated the robustness and adaptability of our Modified MobileNet model, substantiating its high performance not only on our initial dataset but also when exposed to novel, external data. Consequently, these findings suggest promising potential for the model’s implementation in real-world clinical settings, aiding in the quick and accurate diagnosis of skin cancer.

## 4. Discussion

This study is dedicated to developing an efficient, accurate, and automatic method for the diagnosis of actinic keratosis (AK). We improved the lightweight convolutional neural network, MobileNet, and the improved model achieved an accuracy and AUC of 0.9625 and 0.97, respectively, with the shortest training time of only 821 s. This may be related to the characteristics of actinic keratosis. Compared with pathological images of patients with breast cancer, lung cancer, and colorectal cancer, the texture features of skin keratosis are relatively simple. In the feature extraction based on special images with sample textures, large-scale deep CNN models with more convolutional layers tend to overfit when the image dataset is relatively small. Among these models, our model requires less time and cost, making it more favorable for deployment in a clinical environment and promising for better diagnosis of actinic keratosis in the future.

Actinic keratosis is a precancerous skin lesion with a high degree of harm and is easily confused with non-actinic keratosis. Currently, the diagnosis of actinic keratosis mainly relies on doctors’ clinical experience and histopathology, but the diagnosis is difficult and easily confused with other diseases. Therefore, our research results have significant clinical implications. Through our model, doctors can accurately diagnose actinic keratosis in a shorter time, thus starting treatment as soon as possible and reducing the possibility of deterioration of the condition. 

The conventional diagnostic modalities for actinic keratosis primarily rest on visual inspection, clinical acumen, and histopathological analysis. These established methods, although efficacious, come with their unique set of challenges. One key issue is that visual inspection is heavily reliant on clinician’s expertise and is prone to individual variances in judgement. The diagnostic complexity is intensified by the fact that actinic keratosis can be readily mistaken for other skin conditions.

The histopathological examination, considered the definitive gold standard for diagnosis, is inherently invasive, potentially causing discomfort to patients. Moreover, it is labor-intensive and time-consuming, requiring sophisticated laboratory resources and specialized personnel. Such factors contribute to substantial healthcare costs and can potentially restrict diagnostic accessibility in resource-limited settings.

Both visual inspection and histopathological evaluation are subject to numerous subjective factors, including clinician’s experience, patient’s symptomatology, and interpretation of pathological images. The cumulative effect of these constraints may lead to diagnostic delays or inaccuracies, thereby posing a risk to patient outcomes.

Our improved MobileNet model addresses many of these concerns, providing a non-invasive, cost-efficient, and objective alternative for actinic keratosis diagnosis. Capitalizing on the capabilities of machine learning, our method enhances diagnostic precision, efficiency, and accessibility.

Despite the considerable advancements made with the model in this study, it is crucial to contextualize our findings within the wider landscape of deep learning in dermatological diagnostics. As there are few related studies on using deep learning algorithms to diagnose AK, we compared the results of our study with some recent studies of other skin diseases. For instance, Li et al. [30] utilized the deep CNN Inception-V3, achieving an AUC of 0.8000 on 2000 melanoma images, while Codella et al. [31], using a fusion of deep CNNs, sparse coding, and SVM, garnered an accuracy, sensitivity, and specificity of 0.7390, 0.7380, and 0.7430, respectively, on 2624 samples. Despite these successes, deep CNNs have not universally triumphed in skin disease recognition. Mirunalini et al. [32]’s employment of GoogleNet transfer learning on a 2000-sample melanoma dataset from ISIC only yielded an overall accuracy of 0.6580. To further validate our model’s merit, we compared it with studies utilizing traditional machine learning algorithms. Vasconcelos et al. [33]’s color evaluation method, which incorporated feature selection and machine learning classification, achieved identification accuracies of 0.7775 and 0.8138 on two smaller public datasets. Similarly, Ohki et al. [34] employed the traditional RF classifier model, reaching a sensitivity and specificity of 0.7980 and 0.8070 on 1148 skin images.

Our model, drawing inspiration from Wang L et al.’s shallow neural network method, AK-DL (2020), elevates this pioneering framework which prioritized keratosis diagnosis. Their model represented a critical stride in the application of shallow neural networks in dermatology, demonstrating superior performance over deep neural networks. Despite its notable accuracy of 92.5%, the model had room for improvement. This current study introduces an innovative model that offers both computational efficiency and a balanced approach towards diagnostic accuracy and computational cost. Outperforming the AK-DL model and comparable deep learning methods with an accuracy and AUC of 0.9265 and 0.97, our model presents a significant advancement in the field. Our model is improved based on MobileNet, combining the advantages of lightweight convolutional neural networks, including high computational efficiency and low model complexity. At the same time, we made some special designs and optimizations for the characteristics of actinic keratosis. Specifically, we made meticulous adjustments to the structure and parameters of the model to better adapt to the diagnostic task of AK. For example, we optimized the network structure of the model to better adapt to our classification diagnostic task. After parameter debugging, we found the activation function and loss function suitable for this task to further improve the performance of the model. These improvements made our model achieve excellent results in the diagnosis of actinic keratosis.

Training time is one of the important indicators for evaluating model performance. Our model, while maintaining high accuracy, has a training time of only 821 s, much lower than other deep learning models. This is thanks to the lightweight design of MobileNet, whose depthwise separable convolution greatly reduces the computational complexity of the model. In addition, the number of model parameters has also been greatly reduced, making the model easier to deploy on devices. This lightweight model is of great significance for future applications, especially considering embedded devices such as bracelets. With the trend of medical device portability, embedding the model into portable devices can help doctors and patients monitor skin lesions in real time, thereby discovering and treating actinic keratosis earlier.

Despite the excellent performance of our model in recognizing actinic keratosis, there are some limitations in the research. First, our model was trained and tested based on the publicly available HAM10000 dataset, which may limit the generalization ability of the model. In the future, we need to collect more data, including skin images of different races, different age groups, and different stages of lesions to further improve the generalization performance of the model. Secondly, although the training time of our model has been greatly reduced, the training cost needs to be further reduced. We will continue to optimize the model structure, further lightweight the model, and improve the accuracy of the model. These optimizations will make the model more suitable for running on resource-limited devices. Finally, although our research mainly focuses on the development of algorithms, using convolutional layers to extract image details of end-to-end recognition patterns is beneficial for frontend system development, but to apply this technology to actual clinical environments, a lot of system development and deployment work needs to be performed. This includes integration with existing medical devices and systems, as well as compliance with various regulations and requirements in the medical industry. In addition, we also need to work closely with doctors and patients to ensure that our system can meet their needs.

In summary, although our research achieved some initial success, there is much work to be performed. Recently, various novel methods have been developed for the diagnosis of AK, such as Markov chain models [35], Antera 3D technology [36,37], in vivo or in vitro Reflective Confocal Microscopy (RCM) [38,39], and dermatoscope analysis has begun to be used for the diagnosis and post-treatment monitoring of skin diseases. The detection effect of the above methods has been relatively effectively verified in practical applications, but their equipment and technical costs are high. We look forward to further improving the performance of the model, expanding the coverage of the dataset, reducing training costs, and successfully deploying our model in actual clinical environments.

## 5. Conclusions

In this study, we aimed to develop an efficient, automated, and accurate method for diagnosing actinic keratosis. An improved model was developed based on MobileNet, incorporating custom preprocessing features such as Lab Space and Contrast Limited Adaptive Histogram Equalization, and the last few layers of the network structure were modified to suit our auxiliary diagnostic task. At the same time, we used five-fold cross-validation to enhance the stability and generalization ability of the model. Comparative results with other mainstream models demonstrate that our model has higher accuracy and faster speed, which is of significant clinical relevance: This research work contributes to precise, efficient, and automatic auxiliary diagnosis of actinic keratosis. The method has the potential to be embedded in portable medical devices, representing high clinical translational value.

## Figures and Tables

**Table 1:** Comparison of improved MobileNet and mainstream machine learning algorithms.

Models	Accuracy (Mean ± 95% CI)	Sensitivity	Specificity	MCC	Training Time (s)
Modified MobileNet	0.9265 ± 0.0336	0.9205 ± 0.0389	0.9242 ± 0.0621	0.8694 ± 0.0472	821.7351
Unmodified- MobileNet	0.8443 ± 0.0818	0.8133 ± 0.0912	0.8270 ± 0.0708	0.7912 ± 0.0564	1140.9066
ResNet	0.7008 ± 0.0912	0.6514 ± 0.0455	0.8465 ± 0.0931	0.6130 ± 0.0715	3757.0801
GoogleNet	0.8408 ± 0.0348	0.8448 ± 0.0759	0.7703 ± 0.0511	0.8241 ± 0.0956	2918.7124
EfficientNet	0.8536 ± 0.0575	0.9233 ± 0.0508	0.7586 ± 0.0689	0.7967 ± 0.0539	3269.5909
Xception	0.8325 ± 0.0710	0.8317 ± 0.0794	0.7624 ± 0.0690	0.7862 ± 0.0626	2525.3332

**Table 2:** Comparison of improved MobileNet and mainstream machine learning algorithms on the external validation.

Models	Accuracy	Sensitivity	Specificity	MCC
Modified MobileNet	0.9197	0.9088	0.9246	0.8575
Unmodified MobileNet	0.7411	0.6433	0.8465	0.6041
ResNet	0.6986	0.7642	0.6514	0.6249
GoogleNet	0.8400	0.8304	0.7592	0.8312
EfficientNet	0.8763	0.9180	0.7909	0.8277
Xception	0.7631	0.8159	0.6924	0.7363

## Data Availability

The data presented in this study are available in this article.
